# Peer Review of “Data Obfuscation Through Latent Space Projection for Privacy-Preserving AI Governance: Case Studies in Medical Diagnosis and Finance Fraud Detection”

**DOI:** 10.2196/72523

**Published:** 2025-03-12

**Authors:** Reenu Singh

**Affiliations:** 1 Indian Institute of Management Mumbai, Mumbai, India

**Keywords:** privacy-preserving AI, latent space projection, data obfuscation, AI governance, machine learning privacy, differential privacy, k-anonymity, HIPAA, GDPR, compliance, data utility, privacy-utility trade-off, responsible AI, medical imaging privacy, secure data sharing, LSP, artificial intelligence

*This is a peer-review report for “Data Obfuscation Through Latent Space Projection for Privacy-Preserving AI Governance: Case Studies in Medical Diagnosis and Finance Fraud Detection.”*

## Round 1 Review

### Specific Comments

#### Major Comments

1. What was the basis of taking up health care cancer diagnosis and financial fraud for the study [1]? Will latent space projection be an effective method for privacy protection in speech therapy to analyze audio datasets to assist in diagnosing and treating speech-related disorders; in medical imaging video datasets from endoscopy, ultrasounds, and robotic surgeries for diagnostics and artificial intelligence–assisted tools; and in telemedicine to analyze video feeds for remote consultations and diagnoses?
2. The basic structure of the paper is missing. Please follow the guidelines of journal paper writing with distinctly visible sections of Introduction, Method, Result/Findings, Discussion, and Limitations with future scope and conclusion. The introduction, background, and related work should be written cohesively, and all should come under the Introduction heading.
3. The statistical tables are in excess. The tables and values should be talked about in written form. Limit the number of images and tables to 5‐6 or according to the journal guidelines. Use an appendix for the flowchart and any other tabular data that is too lengthy.
4. Explanations of tables and figures should be in paragraph form. Please cite literature where comparative inference and process-specific benefits and drawbacks are mentioned. Examples are Tables 1-5. For writing sections like “Comparative Analysis with Existing Techniques,” all the subparts should be written in paragraphs and discuss the values and analysis only, and put them in their respective paragraphs, removing the tabular data. Please use appendices for excessive tables. Within the body of the research paper, 5‐6 figures and tables are sufficient; the rest should be put in appendices.
5. In “Latency and Performance analysis, part A” and “Performance optimization” are mentions of the literature, which should be present as part of the literature in the Introduction paragraph. Restating the literature again is redundant. Stick to the structure of the journal paper. Please cite references to support the claims, such as “real-time requirements of financial systems” under the section of Real-Time Performance.
6. “Scalability analysis” and other sections: What were the criteria for the choice of datasets for the study for the case studies? What were the data sizes? Give specifications in the first paragraph of respective case studies. Presenting the details about the process of procurement of files, data extraction, limitations in data handling, etc. Are there any limitations in adopting the latent space projection methods?

## Round 2 Review

### General Comments

This paper is highly relevant to health care, particularly in the context of privacy management of data during the analysis of imagery.

### Specific Comments

#### Major Comments

1. The case studies should be written in a more descriptive style. Please reduce the use of numbered or bullet points (in the Introduction, Method, and Result) to align with the formal writing style typically suitable for journal papers.

2. Please rephrase the description of Table 3 (immediately following the table) in a narrative style. This approach enhances the readability of the article.

3. Two figures should not be positioned consecutively. Include some text between Figure 3 and Figure 4. Adjust and reorganize the content to ensure a smooth flow.

#### Minor Comments

4. The titles of tables and figures should be presented as captions. Revise the captions to ensure they do not begin with a verb.
